# Editorial: Transmission dynamics and population genomics of superbug pathogens of public health importance

**DOI:** 10.3389/fcimb.2026.1978910

**Published:** 2026-09-15

**Authors:** Kok Keng Tee, Elijah Ige Ohimain, Ezera Agwu

**Affiliations:** 1 Yunnan Provincial Key Laboratory of Public Health and Biosafety, School of Public Health, Kunming Medical University, Yunnan, China; 2 Yunnan Provincial Key Laboratory of Cross-Border Infectious Diseases Control and Drug Innovation, School of Public Health, Kunming Medical University, Yunnan, China; 3 Department of Microbiology, Niger Delta University, Wilberforce Island, Bayelsa, Nigeria; 4 School of Public Health, University of Port Harcourt, Choba, Rivers, Nigeria; 5 Department of Microbiology and Parasitology, School of Medicine and Pharmacy, College of Medicine and Health Sciences, University of Rwanda, Butare, Rwanda

**Keywords:** bacterial genomics, bacterial infectins, disease transmission dynamics, drug resistance, epidemiological monitoring, molecular epideiology, whole genome sequencing (WGS)

## Introduction

Antimicrobial-resistant and high-risk bacterial pathogens are shaped by processes operating across multiple biological and epidemiological scales: genetic variation within pathogen populations, acquisition and dissemination of resistance determinants, transmission within healthcare and community settings, and interactions with susceptible hosts (Sati et al., 2025). Understanding any one of these dimensions in isolation provides only a partial view of why particular pathogens emerge, persist, spread, or cause disproportionate clinical and public-health burdens (GBD 2021 Antimicrobial Resistance Collaborators, 2024). This Research Topic was conceived to bring these levels together, with particular emphasis on transmission dynamics and population genomics. Its 16 contributions span genomic epidemiology, antimicrobial resistance, outbreak investigation, clinical epidemiology, host–pathogen interactions, and surveillance across healthcare, population, and community settings. Collectively, they can be considered through four complementary themes.

## Genomic diversity, evolution, and antimicrobial resistance

Population genomics provides a framework for distinguishing successful lineages, tracking their geographical persistence, and identifying genetic traits that may contribute to adaptation, resistance, or virulence. Humberto et al. characterized major *Acinetobacter baumannii* lineages in Mexico and identified multiple international clones with distinct β-lactamases, illustrating how genomic data can reveal both lineage diversity and persistence across locations and time. Li et al. extended this population-level perspective to carbapenem-resistant hypervirulent *Klebsiella pneumoniae* (CR-hvKP) in China, highlighting the convergence of antimicrobial resistance and hypervirulence within diverse genetic backgrounds.

Genomic epidemiology can also help distinguish evolutionary emergence from the resurgence of established pathogen populations. Wang et al. showed that the post-pandemic resurgence of *Mycoplasma pneumoniae* in Beijing in 2023 was associated with pre-existing strains rather than a newly emerged variant, while identifying regional genomic differentiation and changing macrolide-resistance patterns. Liu et al. combined an unusual clinical presentation of pneumococcal pericarditis with broader genomic screening to place multidrug-resistant ST876 serotype 14 *Streptococcus pneumoniae* within a wider evolutionary context. At the boundary between recognized and emerging human pathogens, Zhao et al. used whole-genome sequencing and phylogenetic analysis to characterize a rare human *Streptococcus parasuis* isolate, underscoring the value of genomic contextualization when the pathogenic, resistance, and zoonotic significance of an organism remains incompletely defined.

Together, these studies illustrate a common principle: antimicrobial resistance and pathogenic potential are properties of evolving pathogen populations rather than static species labels. Population genomics is therefore most informative when it captures lineage replacement, resistance acquisition, virulence-associated traits, and geographical structure through time.

## From resistance evolution to outbreak transmission

The public-health importance of resistance depends not only on its emergence but also on its capacity to disseminate. Ma et al. traced the evolutionary trajectory of an IncP-2 plasmid co-harboring *bla*_IMP-45_ and *bla*_VIM-1_ during an outbreak of ST313 carbapenem-resistant *Pseudomonas aeruginosa*. By integrating whole-genome sequencing, plasmid characterization, phylogenetic analysis, and conjugation experiments, the study illustrates that the epidemiological units of resistance dissemination can include mobile genetic elements as well as bacterial clones.

At the healthcare level, Song et al. investigated a suspected cluster of carbapenem-resistant *Acinetobacter baumannii* lower respiratory tract infections in a cardiovascular surgical intensive care unit. Integration of epidemiological investigation, environmental sampling, and whole-genome sequencing showed that the clustered cases did not constitute a nosocomial infection outbreak, while identifying genetically related patient and environmental isolates and potential transmission-risk points. These studies demonstrate why genomic data are most informative when integrated with conventional epidemiology: whole-genome sequencing can not only identify plausible transmission relationships but also distinguish genetically unrelated infections that might otherwise be interpreted as part of the same outbreak.

More broadly, resistance can disseminate simultaneously across several levels – from genes and transposons to plasmids, bacterial clones, patients, and healthcare networks. Understanding these interconnected levels is central to moving from retrospective outbreak description towards more effective interruption of transmission.

## Host susceptibility, clinical phenotype, and disease outcomes

The clinical consequences of bacterial infection reflect interactions among pathogen traits, host susceptibility, and the clinical context in which infection occurs. Several contributions in this Research Topic move beyond pathogen population structure to examine factors associated with colonization, disease phenotype, and outcome. Gu et al. compared MRSA pneumonia in patients with and without COVID-19. Patients with COVID-19/MRSA co-infection were older, had greater comorbidity burdens and more severe pneumonia, and had higher mortality than patients with MRSA pneumonia without COVID-19 (44% vs. 18%). Notably, the two groups did not differ significantly in the distribution of antimicrobial-resistance or virulence-factor genes, underscoring that clinical outcome cannot be inferred from pathogen genotype alone.

Host factors may also influence earlier stages of the infection continuum. Using NHANES 2001–2004 data, Chen et al. found that higher serum total protein and globulin concentrations were inversely associated with concurrent nasal MRSA colonization after multivariable adjustment and propensity-score matching. Because the study was cross-sectional, these findings identify associations rather than causal protective effects, but they illustrate how host characteristics may shape colonization risk.

The relationship between microbial phenotype and clinical presentation was further explored by Jiang et al., who compared hypervirulent and classical *Klebsiella pneumoniae* isolates from a tertiary hospital in southern China. Hypervirulent isolates were more often associated with bacteraemia and less often with urinary tract infection, whereas multidrug resistance was more frequent among classical isolates; whole-genome sequencing also identified an ST11-K64 hypervirulent carbapenem-resistant isolate. These findings highlight that virulence and antimicrobial resistance are intersecting but non-equivalent properties. In a different clinical setting, Li et al. combined conventional epidemiological analyses with interpretable machine-learning models in a retrospective cohort of 5,862 individuals, identifying *Helicobacter pylori* infection as independently associated with peripheral atherosclerosis and as an influential predictor in risk models. The analysis supports an association and potential value for risk stratification, rather than establishing a causal vascular effect of infection.

Finally, Feng et al. examined clinical determinants of prolonged osteomyelitis in a single-centre cohort of 704 patients. Fracture-related infection, femoral involvement, pathogen-positive culture, and *Staphylococcus aureus* isolation were associated with a prolonged disease course. Together, these studies emphasize that the burden of high-risk bacterial pathogens is ultimately expressed through interactions among microbial characteristics, host factors, comorbidity, infection site, and clinical management.

## Extending surveillance across healthcare, population, and community settings

Surveillance links observations in individual patients and isolates to population-level understanding. The contributions in this theme illustrate complementary surveillance approaches across molecular, healthcare, and community settings. Li et al. analysed 1,907 clinical samples collected from 13 tertiary hospitals to characterize P1 adhesin genotypes of *Mycoplasma pneumoniae* from 2017 to 2019. P1–1 remained dominant, whereas the proportion of P1–2 increased among children over time; importantly, P1 genotype was not associated with disease severity. The study demonstrates how molecular surveillance can detect shifts in circulating genotypes even when those changes do not translate directly into differences in clinical severity.

Longitudinal healthcare surveillance provides a different epidemiological perspective. Liang et al. analysed hospital records from Guizhou Province from 2015 to 2023 and identified 6,944 *Acinetobacter baumannii*-positive events among 460,620 patients. Of the positive cases, about 46% occurred in ICU patients, while older age, male sex, and seasonal patterns were associated with positivity. Such data are useful for identifying groups and settings with a higher observed burden, although their retrospective design does not by itself establish causal drivers of infection.

Genomic surveillance can add resolution where conventional systems are incomplete. Rosato et al. analysed 61 invasive group A *Streptococcus* isolates collected at an Italian tertiary hospital between 2016 and 2024, revealing substantial emm-type diversity and detecting the M1UK sub-lineage from 2018 without evidence of a marked post-pandemic upsurge. Their findings illustrate both the value of local genomic data and the limitations of interpreting pathogen dynamics in a fragmented surveillance landscape, supporting the need for coordinated national genomic surveillance.

Surveillance also needs to extend beyond healthcare facilities. Sultan et al. sampled frequently touched surfaces in 258 public-transport vehicles in Harar, Ethiopia; pathogenic bacteria were recovered from 74.4% of vehicles and multidrug resistance was detected in 22.5% of isolates. These findings identify public transport as a potential community interface for exposure to resistant bacteria, but environmental detection should not be equated with demonstrated person-to-person transmission. Taken together, these studies show that surveillance is most informative when microbiological and genomic data are interpreted alongside time, place, population, and exposure context.

## Conclusion

Collectively, the studies in this Research Topic show that the public-health impact of antimicrobial-resistant and high-risk bacterial pathogens emerges from interconnected processes spanning pathogen evolution, resistance acquisition, transmission, host susceptibility, and surveillance context. Whole-genome sequencing has increased the resolution with which lineages, resistance determinants, and suspected transmission events can be investigated, but genomic data are most informative when integrated with clinical, epidemiological, and environmental evidence. Strengthening longitudinal and genomic surveillance across healthcare and community settings, while linking molecular findings to patient outcomes and exposure networks, will be important for detecting emerging high-risk lineages and translating pathogen genomics into effective infection-prevention and antimicrobial-stewardship strategies.
